# When Food Meets Man: the Contribution of Epigenetics to Health

**DOI:** 10.3390/nu2050551

**Published:** 2010-05-25

**Authors:** Emma De Fabiani, Nico Mitro, Federica Gilardi, Andrea Galmozzi, Donatella Caruso, Maurizio Crestani

**Affiliations:** 1 “Giovanni Galli” Laboratory of Biochemistry and Molecular Biology of Lipids and Mass Spectrometry, Department of Pharmacological Sciences, Università degli Studi di Milano, Via Balzaretti 9, 20133 Milano, Italy; Email: nico.mitro@unimi.it (N.M.); federica.gilardi@unimi.it (F.G.); andrea.galmozzi@unimi.it (A.G.); donatella.caruso@unimi.it (D.C.); maurizio.crestani@unimi.it (M.C.); 2 “Giovanni Armenise-Harvard Foundation” Laboratory, Department of Pharmacological Sciences, Università degli Studi di Milano, Via Balzaretti 9, 20133 Milano, Italy; 3 “Giovanni Galli” Center for the Characterization and Safe Use of Natural Products, Università degli Studi di Milano, Via Balzaretti 9, 20133 Milano, Italy

**Keywords:** fasted-to-fed cycle, gluconeogenesis, bile acid synthesis, cholesterol metabolism, chromatin, histone deacetylases, transcriptional regulation, resveratrol, sirtuins

## Abstract

Post-translational modifications of chromatin contribute to the epigenetic control of gene transcription. The response to food intake and individual nutrients also includes epigenetic events. Bile acids are necessary for lipid digestion and absorption, and more recently have emerged as signaling molecules. Their synthesis is transcriptionally regulated also in relation to the fasted-to-fed cycle, and interestingly, the underlying mechanisms include chromatin remodeling at promoters of key genes involved in their metabolism. Several compounds present in nutrients affect gene transcription through epigenetic mechanisms and recent studies demonstrate that, beyond the well known anti-cancer properties, they beneficially affect energy metabolism.

## 1. Introduction

The increasing understanding of relevant biological processes and the development of sensitive and high-throughput technical approaches to study important cellular functions have allowed us to investigate in depth the molecular mechanisms underlying the effects of nutrients and individual compounds in mammals. A number of dietary components exert their beneficial effects on human health by modulating the expression of genes involved in the pathogenesis and/or in the protective mechanisms relative to epidemiologically relevant diseases (e.g., cancer, cardiovascular diseases). In this respect, the downstream effects of post-translational modifications of histone proteins and other DNA-interacting proteins are emerging as crucial aspects contributing to the phenotypic response to food intake and to individual nutrients.

The general purpose of this review is to summarize experimental observations documenting that chromatin remodeling in specific regions: (i) participates in the metabolic adaptation to the fed-fasting cycle, and (ii) is influenced by compounds either found in food or derived from metabolic transformation of food components.

To this aim, basic concepts in epigenetics, chromatin dynamics, and the post-translational modifications through which chromatin is remodeled, will be reviewed. Particular emphasis will be given to histone deacetylases and sirtuins that have emerged as key factors in the remodeling of nucleosomes, and regulators of the association and activation state of transcriptional factors and cofactors to target promoters. Then, we will describe how epigenetic mechanisms contribute to the physiological response of the “eating-not eating” cycle, focusing in particular on gluconeogenesis and bile acid synthesis, two metabolic pathways that are modulated at the transcriptional level through similar regulatory mechanisms in response to hormonal stimuli. Furthermore, we will discuss how bile acids, terminal products of cholesterol synthesis and signaling molecules, by promoting the association of several chromatin remodeling proteins, modulate gene transcription. Finally we will present an overview of experimental studies reporting the molecular effects of dietary regimens or dietary supplementations that, in different ways, affect chromatin structure and gene transcription, thus possibly benefiting the treatment of metabolic disorders.

## 2. Basic Concepts in Epigenetics

Epigenetics can be defined as any inheritable influence on gene activity that does not involve a change in DNA sequence. In all eukaryotic cells, genomic DNA is folded and compacted by histone and non-histone proteins to form nucleosomes, and a further structured and dynamic form, generally referred to as chromatin. DNA packaging must be considered as a way to store and retrieve information since it represents a dynamic switch between transcriptional “on-off” states that result in gene activation and gene silencing. The term euchromatin refers to accessible and transcriptionally active genomic regions, while heterochromatin is composed of highly condensed and transcriptionally less active genetic material.

**Figure 1:**
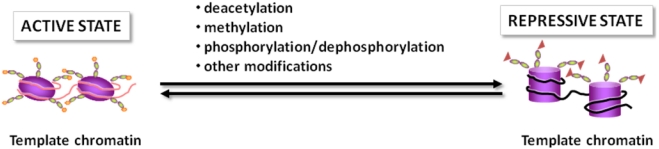
The dynamics of DNA packaging. Post translational modifications of histone and non-histone proteins impose changes in the nucleosomal organization and represent the dynamic switch between transcriptional “on-off” states of chromatin.

In general, the epigenetic control is accomplished by means of two major mechanisms: methylation of cytosine residues in the DNA sequence and modifications of histones and other chromatin-associated proteins. Referring to the latter mechanism, it has long been known that histone tail domains can be covalently modified by acetylation, phosphorylation, methylation and ADP-ribosylation at specific amino acid positions (Figure 1) in a way that regulates the contacts with the underlying DNA [1]. More recently, it has become evident that acetylation, phosphorylation, and newly discovered modifications (e.g., ubiquitination and sumoylation) also occur at the level of other chromatin associated proteins, transcription factors, transcriptional co-activators and co-repressors, thus modulating their function, and ultimately regulating gene transcription (Figure 1).

### 2.1. The Histone Code

The term “histone code” was proposed in 2001 by Jenuwein and Allis [2] to define a marking system consisting of amino-terminal modifications of histone proteins. These modifications are then read by effector molecules, for example co-activators and co-repressors of gene transcription, and translated into biological functions, including transcription. It has been suggested that these chromatin marks might function in a combinatorial manner, thus increasing their indexing potential or capacity. Altogether, this marking system considerably extends the information potential of the genetic code, and represents an important level of regulation in response to endogenous and exogenous stimuli.

### 2.2. Chromatin Remodeling Enzymes

As mentioned above, histones and chromatin-associated proteins are the targets of multiple post-translational modifications and several enzymes act on chromatin by introducing different post-translational modifications at specific amino acids. These modifications include methylation of arginine residues; methylation, acetylation, ubiquitination, ADP-ribosylation, and sumoylation of lysines; and phosphorylation of serines and threonines [3].

A single histone protein presents several residues that can undergo post-translational modification giving rise to a variety of modified products. An example of this complexity is given by the peptide mass fingerprinting of core histones by mass spectrometric analysis that is able to detect methylation, acetylation, phosphorylation and ubiquitination. This analysis revealed the presence of 13 modification sites in histone H2A, 12 modification sites in histone H2B, 21 modification sites in histone H3, and 14 modification sites in histone H4 [4]. Each site can either be unmodified or modified. In addition, some lysine residue can either be methylated or acetylated and there are three different possibilities for each methylated site (mono, di or tri). Therefore, histone proteins may undergo an extremely large number of possible combinations of modifications.

The post-translational modification state of chromatin is the result of the balanced activity of several enzymes. For instance, the acetylation state of histones is the result of the activity of acetyltransferases and deacetylases.

From the functional point of view, acetylation of histone H3 and H4 is associated with active transcription and commonly referred to as euchromatin modifications. This is most likely due to the fact that acetylation loosens inter- or intra-nucleosomal DNA-histone interactions and, at the same time, affects recognition and protein-protein interactions. On the other side, evidence suggests that methylation at lysine 9 of histone H3 is a mark for transcriptionally silent chromatin [5]. This modification can be catalyzed by specific enzymes, such as histone methyl transferase G9a, which islocalized exclusively in euchromatic regions [6].

### 2.3. Histone Deacetylases

Addition of acetyl groups is one of the most widespread modifications of histones. Acetylation at ε-amino groups of lysine residues in histone tails neutralizes their positive charges, thereby relaxing chromatin structure. Furthermore, acetylated histones also serve as binding sites for bromodomain proteins, often acting as transcriptional co-activators. Histone acetylation occurs at multiple lysine residues; it is usually carried out by a variety of histone acetyltransferase complexes (HATs) and is reversed through the action of histone deacetylases (HDACs) [2,7].

HDACs do not bind DNA directly; in contrast, through the interaction with transcriptional activators and co-activators, they are recruited to target genes and are assembled in large multiprotein transcriptional complexes. Thus, the specific contribution of HDACs to the regulation of gene transcription depends on the cell type and on the availability of interacting proteins.

HDACs represent a large super family of proteins whose members are grouped in different classes (Table 1) [8]. Class I HDACs are ubiquitously expressed and found predominantly in the nuclear compartment. They bear a conserved deacetylase domain and are active on histone substrates. The catalytic domain consists of a narrow, tube-like pocket, at the bottom of which a zinc cation is positioned. In cooperation with two histidine-aspartate charge relay systems, the zinc cation facilitates the deacetylation reaction.

Class IIa HDACs carry a large N-terminal with conserved binding sites for the transcription factor myocyte enhancer factor 2 (MEF2) and the chaperone protein 14-3-3. Upon phosphorylation by means of different kinases, class IIa HDACs bind 14-3-3 and translocate from the cytoplasm to the nucleus [9,10,11,12]. Class IIa HDACs possess only minimal histone deacetylase activity, a behavior that can be explained with the fact that a key tyrosine residue in the catalytic domain is substituted with a histidine in class IIa HDACs [13]. A critical point in the evaluation of histone deacetylase activity is the physical interaction between class I and class II HDACs. In fact, it was shown that HDAC4 (class IIa) recruits pre-existing enzymatically active complexes containing HDAC3 (class I) and nuclear co-repressors (NCo-R and SMRT), and that these protein-protein interactions are crucial in the transcriptional repression mechanisms involving histone deacetylation [14]. These evidences suggest that class II HDACs behave as bridging molecules with little enzymatic activity, however at present it cannot be excluded that these enzymes act on other substrates that are still unknown.

The most characterized member of class IIb HDACs is HDAC6, a cytoplasmic protein that acts on non-histone substrates such as cytoskeletal proteins and transmembrane proteins [8].

Finally, HDAC11 is the only member of class IV whose functions are still elusive [8].

### 2.4. Beyond Histones

As briefly mentioned above, histones are not the only proteins whose biological activities are modulated by the acetylation state at lysine residues [15]. In particular, acetyltransferases themselves, such as the transcriptional coactivators p300 and CREB (cyclic AMP response element-binding protein) binding protein (CBP), are heavily acetylated. Acetylation at lysine residues occurs also at the level of other transcription factors and cofactors. In addition, also cytoplasmic proteins present acetylation sites. 

Sirtuins were initially discovered as silencing factors and longevity-linked proteins in lower organisms. The discovery that they act as NAD^+^-dependent histone deacetylases [16] represented a significant breakthrough that allowed appreciating the multiple roles played by these enzymes in patho-physiology. Besides on histones, sirtuins act on a variety of acetylated substrates with different functions. SIRT1 deacetylates transcription factors and co-activators such as p53, nuclear factor κB, proteins belonging to the forkhead box type O (FOXO) family, the nuclear receptors peroxisome proliferator activated receptor (PPAR) γ, farnesoid X receptor (FXR), PPAR γ-coactivator 1 (PGC-1) α; but it also acts on enzymes such as acetyl-CoA synthetases, or on structural proteins such as α-tubulin and heat shock proteins [17,18].

**Table 1:** The mammalian histone deacetylase superfamily. The list does not include class III HDACs, a term sometimes used to indicate sirtuins, a distinct group of deacetylases.

Class	Protein domains	Members
Class I	Deacetylase catalytic domain	HDAC1
	Phosphorylation sites (serine residues) at C terminus	HDAC2
		HDAC3
		HDAC8
Class IIa	Deacetylase catalytic domain	HDAC4
	Phosphorylation sites (serine residues) at N terminus	HDAC5
	Myocyte enhancer factor binding sites	HDAC7
	Binding sites for 14-3-3 chaperone protein	HDAC9
Class IIb	Deacetylase catalytic domain	HDAC6
	Zinc finger domain or leucine rich region	HDAC10
Class IV	Deacetylase catalytic domain	HDAC11

## 3. Epigenetic Regulation of Metabolic Pathways

In recent years, chromatin remodeling events have been demonstrated to be widely involved in the transcriptional control of metabolic homeostasis. In parallel with the advances in the understanding of the role of chromatin dynamics in patho-physiology, the ability of specific foods and of dietary regimens to exert beneficial effects through epigenetic mechanisms has been taken into consideration and investigated in more depth. The hypothesis is that either the response to food intake and to individual compounds present in or deriving from foods may affect chromatin structure.

Actually, a large number of studies have clearly demonstrated that some dietary components affect gene transcription, through multiple mechanisms. To mention few examples, fatty acids can act as ligands of membrane and nuclear receptors, thus regulating intracellular signaling and gene expression [19,20], while polyphenols, present in a large number of food sources, exhibit anti-inflammatory activities by interfering at multiple levels with the activation cascade of nuclear factor-κB, a key regulator of the inflammatory response [21].

Deciphering the impact of dietary regimens (e.g., fast-feeding cycle, calorie restriction) or of specific nutrients (e.g., vegetables, fibers) on epigenetics will provide essential information for the “evidence-based” assessment of their benefits on human health.

### 3.1. The Fed-Fasting Cycle: the Role of Bile Acids

Bile acids possess multiple functions. Due to their amphipathic nature, through the formation of mixed micelles, they promote solubilization and transport of lipids in an aqueous environment, thus actively contributing to intestinal digestion and absorption of lipids. Primary bile acids are produced in the liver from cholesterol through a complex and highly regulated pathway [22]. The first reaction in this pathway, the hydroxylation of cholesterol at the 7α position, is catalyzed by the enzyme cholesterol 7α-hydroxylase (*CYP7A1*) and represents the rate-limiting step of the whole pathway.

**Figure 2:**
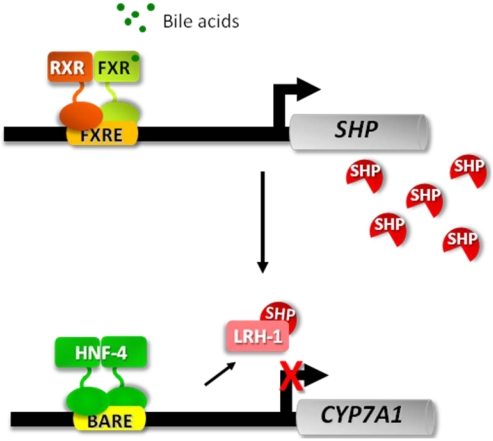
Model describing the molecular basis for feedback regulation of bile acid synthesis. The scheme is based on the findings reported by Goodwin *et al.* and Lu *et al.* [23,24]. It is worth noting that the functions of liver receptor homolog 1 (LRH-1) were revisited more recently and it was concluded that, although this orphan nuclear receptor contributes to bile acid homeostasis, its role in the regulation of *CYP7A1* gene is unexpectedly negligible, at least in the mouse [25].

A major breakthrough in our understanding of bile acid biology came in recent years with the discovery that bile acids are the physiological ligands of a nuclear receptor, the farnesoid X receptor (FXR), and that bile acid-dependent activation of FXR underlies the feedback regulation exerted by bile acids on their own synthesis [23,24]. According to the proposed model, bile acid-activated FXR binds a responsive element in the promoter of small heterodimer partner (SHP) that acts as a transcriptional repressor at the promoters of target genes, such as *CYP7A1* (Figure 2).

**Figure 3:**
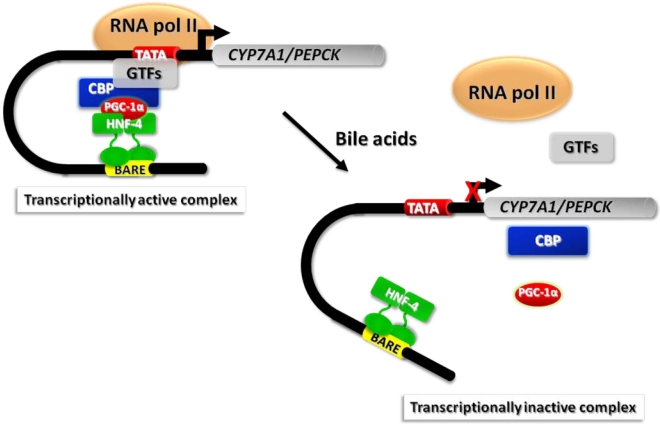
Model describing the mechanism by which bile acids disrupt the formation of a transcriptionally active complex at the promoters of *CYP7A1* and *PEPCK*, the key genes in bile acid synthesis and gluconeogenesis, respectively. Hepatocyte nuclear factor (HNF)-4 bound to a sequence named BARE (bile acid responsive element) interacts with the co-activators PGC-1α and CBP. These protein-protein interactions may promote DNA bending and formation of a multiprotein complex containing general transcription factors (GTFs) and RNA polymerase (RNA pol) II. Bile acids interfere with the interactions between HNF-4 and co-activators and with the association of RNA pol II at the *CYP7A1*/*PEPCK* promoters causing reduction of gene transcription.

Although the FXR pathway is responsible for most of the biological effects exerted by bile acids on gene transcription, the analysis of *shp*-deficient mice indicated that bile acids down-regulate their synthesis, and particularly the *CYP7A1* gene, also by means of additional mechanisms [26,27]. In this regard, we showed that bile acids repress the transactivation potential of hepatocyte nuclear factor (HNF)-4α, a potent activator of *CYP7A1* transcription, in an FXR/SHP-independent manner [28,29]. Furthermore, we found that bile acids interfere with the recruitment of co-activator proteins, such as PGC-1α and CBP, to the *CYP7A1* promoter in the native context of chromatin (Figure 2) [29]. Investigating whether a similar regulatory mechanism involving chromatin remodeling could apply to other genes transactivated by HNF-4α, we found that bile acids repress the expression of the gene encoding the phospho*enol*pyruvate carboxy kinase (*PEPCK*), one of the key enzymes in gluconeogenesis, through a mechanism highly similar to that described for *CYP7A1* [29] (Figure 3). 

A large fraction (~90%) of bile acids is reabsorbed by the intestinal mucosa and returns to the liver through the entero-hepatic circulation. Therefore, in the post-prandial phase, a high amount of bile acids reaches the liver through the portal vein. In contrast, in the fasting condition, the circulating levels of bile acids drop significantly. A well known hallmark of the fasted state is the raise in glucagon plasma level that triggers a signaling cascade in target tissues, particularly the liver, associated with increased intracellular levels of cyclic-AMP (cAMP) that are responsible for the metabolic adaptation to fasting. The *PEPCK* gene is one of the most regulated genes in the fasted-to-fed cycle, being highly expressed in fasting when the gluconeogenesis rate is high, and dramatically repressed in the postprandial condition. Based on cellular studies demonstrating that both *CYP7A1* and *PEPCK* are regulated in a similar fashion by opposing stimuli such as bile acids and cAMP, the first mimicking postprandial conditions and the latter the fasted state [29], we investigated whether this regulation may also be working *in vivo*. In fact, we found that fasted mice express higher levels of *CYP7A1* and *PEPCK* in comparison to fed animals [29]. Based on these evidences, we propose that the fasted-to-fed cycle regulates apparently unrelated metabolic pathways (bile acid synthesis and gluconeogenesis) in a coordinated fashion.

**Figure 4:**
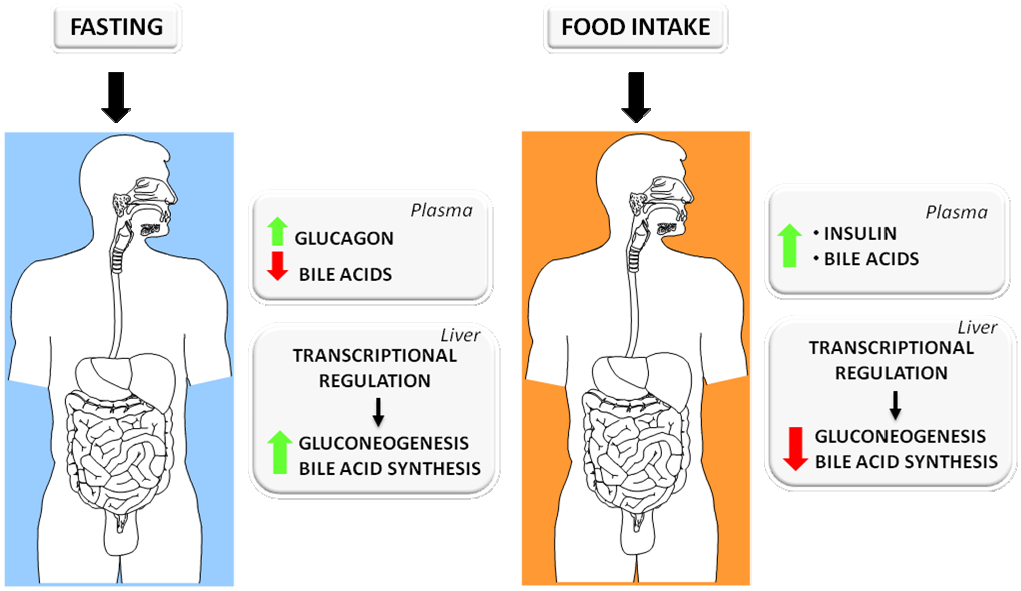
Metabolic changes occurring in the fasted-to-fed cycle. In the fasted state, plasma levels of glucagon are high while the levels of bile acids are low. These signals contribute to the regulation of gene transcription, and the downstream effects are up-regulation of gluconeogenesis and bile acid synthesis. In the fed state, there is a rise of insulin plasma levels and an increase of bile acids returning to the liver from the intestine. Both signals affect gene transcription and, in particular, bile acids repress both *CYP7A1* and *PEPCK*, thus contributing to the repression of gluconeogenesis and bile acid synthesis.

After a prolonged fasting period, the transcription of *CYP7A1* and *PEPCK* increases; probably in response to the stimulation by the glucagon/cAMP cascade and to the concomitant decrease in the concentration of bile acids returning to the liver. On one hand, this may help to prepare the gastrointestinal tract for the digestion and absorption of fats in a subsequent meal and, on the other hand, to increase hepatic glucose production in order to buffer the falling plasma concentration of glucose during the fasted state. Conversely, in the fed state, as the concentration of bile acids fluxing through the entero-hepatic circulation increases, the reduction of *CYP7A1* and *PEPCK* transcription may be secondary to the drop in the glucagon level and to the direct inhibition elicited by bile acids, which are massively secreted into the duodenum and return to the liver at higher concentrations than during a prolonged fasting period (Figure 4).

### 3.2. Sirtuins: Key Players in the Fed-Fasting Cycle and Calorie Restriction

In mammals, sirtuins regulate a variety of functions, from the control of cellular stress to energy metabolism [30]. The first indication that sirtuins, in particular SIRT1, are involved in the metabolic control in mammals came from the elegant studies of P. Puigserver and coworkers, who were investigating the molecular mechanisms responsible for the adaptive metabolic response to fasting. Reduced availability of nutrients, such as that experienced in fasting and calorie restriction, is reflected at the cellular level by a decrease of oxidative pathways and, consequently, by an increase of NAD^+^/NADH ratio. Rodgers *et al*. found that in the fasted state, SIRT1 is induced in the liver and deacetylates PGC-1α at specific lysine residues in an NAD^+^-dependent manner [31]. Deacetylated PGC-1α more actively transactivates the transcription of target genes, particularly those involved in gluconeogenesis [31]. In line with these observations, the knock-down of SIRT1 in the liver leads to reduced glucose production and fatty acid oxidation in the liver, under fasting conditions [32]. These metabolic changes are linked to decreased expression of genes responsible for gluconeogenesis and fatty acid oxidation [32]. These studies clearly demonstrate that SIRT1 is a key regulator of metabolic adaptation to nutrient deprivation and that PGC-1α mediates most of SIRT1 effects. Mitochondria play a central role in metabolic homeostasis and can be considered preferential targets of both SIRT1 and PGC-1α. In fact, the mitochondrial respiratory chain is the main contributor to the NAD^+^/NADH equilibrium, oxidative pathways, *i.e.*, the Krebs cycle and β-oxidation of fatty acids, are localized in mitochondria, and finally, PGC-1α promotes the transcription of mitochondrial genes and mitochondrial biogenesis [33].

### 3.3. Bile Acid-Induced Post-Translational Modifications of Histones and Chromatin Remodeling

The atypical orphan nuclear receptor SHP, as mentioned above, is a crucial player in the signaling pathway by which bile acids regulate several aspects of cholesterol and bile acid metabolism and transport. The simple model proposed initially (Figure 2), according to which bile acid-activated FXR promotes the transcription of SHP, represented a starting point for subsequent investigations that allowed disclosing the contribution of chromatin dynamics to bile acid-sensitive regulatory cascades.

The analysis of the *CYP7A1* promoter showed that bile acids do not cause dramatic structural changes such as nucleosome sliding or disruption [34,35]. However, bile acid treatment results in decreased accessibility of DNA in nucleosome cores to endonucleases in euchromatin, including the *CYP7A1* promoter [34,35,36], indicating a remodeling of the chromatin. Based on the knowledge that chromatin can undergo remodeling driven by ATP hydrolysis, Kemper *et al*. demonstrated that the bile acid-induced SHP is recruited to the *CYP7A1* promoter in association with the repressive complex Swi/Snf that contains the ATPase Brm [35,37]. In addition, SHP promotes the association of a co-repressor complex containing two other chromatin modifying enzymes, HDAC1 and HDAC2 [35,36]. Indeed, the interaction of SHP with histone proteins, in particular with histone H3, is highly influenced by post-translational modifications. In fact, SHP associates with non acetylated/methylated histone H3 and acetylation of histone H3 at lysine 9 prevents this interaction [36]. SHP also induces the recruitment of the G9a methyltransferase [34,36] and the resulting modifications at lysine 9 of histone H3, that is, methylation by G9a and deacetylation by HDACs are interdependent and necessary for the recruitment of the Swi/Snf-Brm complex, which is essential for SHP-mediated transcriptional silencing of the *CYP7A1* gene [34].

As mentioned above, bile acids are able to trigger SHP-independent mechanisms that contribute to the feedback regulation of *CYP7A1*, especially in the initial phase when bile acid-induced SHP protein has not been synthesized yet. Therefore, we investigated the changes imposed by bile acids to chromatin structure in this lag of time, analyzing the composition of the transcriptional complex at the *CYP7A1* promoter [38]. While the co-activators PGC-1α and CBP dissociate from the promoter in bile acid treated liver cells, several co-repressor factors are recruited. In parallel we observed dissociation of RNA polymerase II either from the promoter or from the 3’untranslated region of the CYP7A1 gene. Interestingly, we also found a reduced phosphorylation of RNA polymerase II at the serine-2 residue, which results in a less transcriptionally active enzyme. The structural changes of chromatin also involve the acetylation state; in fact we found that bile acids induce hypoacetylation of histone 4, most likely as a result of a sequential recruitment of several histone deacetylases, HDAC3 and HDAC7 in a first phase, and then HDAC1. In line with these observations, the treatment with HDAC inhibitors prevented the inhibitory effect of bile acids on the mRNA levels of *CYP7A1* of liver cells, and derepressed the expression of the enzyme in mice. To assess the contribution of specific HDACs in this regulatory mechanism, we knocked down the HDAC isoforms recruited in the repressive complex at the *CYP7A1* promoter by RNA interference and we found that only the silencing of HDAC7 prevents the inhibition of *CYP7A1* expression by bile acids. Notably, HDAC7 belongs to class II, and its sub-cellular localization depends on its phosphorylation state, since it is sequestered in the cytoplasm when phosphorylated. We observed that bile acids promote the nuclear translocation of HDAC7 and that calyculin, a phosphatase inhibitor, prevents the effects of bile acids on HDAC7 translocation and the feedback on *CYP7A1* transcription.

**Figure 5:**
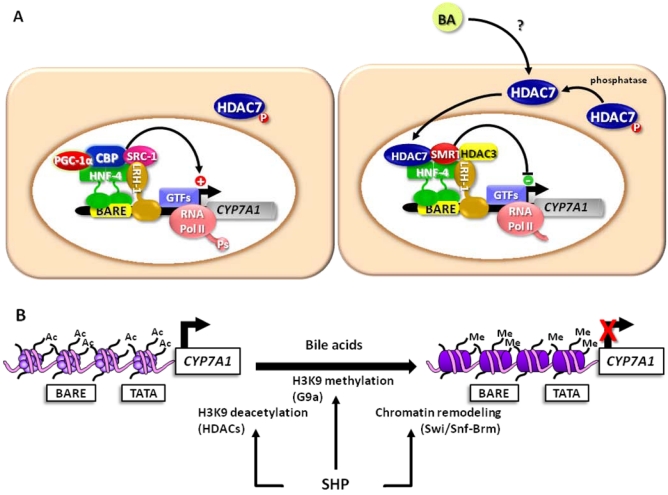
The scheme depicts the role of chromatin remodeling enzymes in the transcriptional regulation of *CYP7A1*. In untreated hepatocytes, a transcriptionally active complex is assembled at the bile acid responsive element (BARE) (A). In addition to the factors reported in Figure 4, other proteins contribute to the transactivation, including liver receptor homolog (LRH)-1 and steroid receptor coactivator (SRC)-1. RNA pol II is phosphorylated and becomes more transcriptionally active. HDAC7 is sequestered in the cytoplasm. In these conditions, the arginine residues of histones, in particular histone H3, are acetylated (B). In the presence of bile acids, in a first phase, HDAC7 translocates to the nucleus and associates with the *CYP7A1* promoter (A). The recruitment of other factors, HDAC3 and the silencing mediator for retinoid and thyroid-hormone receptors (SMRT), contributes to the formation of a repressive complex. By inhibiting phosphatases with calyculin, the bile acid-induced translocation of HDAC7 is blocked and the bile acid-induced inhibition of *CYP7A1* is prevented. On the other hand, bile acids induce the synthesis of SHP that acts as a repressor as depicted in Figure 2. In addition, SHP interacts with HDACs and G9a methyltransferase, which deacetylate and methylate, respectively, arginine residues of histone proteins (B). SHP also promotes the recruitment of the Swi/Snf-Brm complex that drives ATP-dependent chromatin remodeling, which further represses *CYP7A1* transcription (B).

The chromatin remodeling events occurring in the feedback regulation of *CYP7A1* are summarized in Figure 5.

As mentioned above, FXR is one of the initial targets of bile acid signaling and besides up-regulating SHP, it positively modulates the expression of a number of genes involved in bile acid metabolism and transport. 

Consistent with the general function of nuclear hormone receptors, FXR participates in post-translation modifications of chromatin, therefore the recruitment of co-activators that display acetyltransferase activity by activated FXR is not a novel finding.

Other post-translational modifications, such as methylation of histones H3 and H4 following nuclear receptor activation, have emerged more recently. In relation to FXR, it was shown that this nuclear receptor, once activated by bile acids, associates with co-activator-associated arginine methyltransferase 1 (CARM1) at the gene locus of bile salt export pump (BSEP), a well-established FXR-target gene [39]. Enrichment of CARM1 leads to an increase of methylation at lysine 17 and of acetylation at lysine 9 of histone H3 in correspondence of the FXR binding element of BSEP gene, although the interdependence between histone H3 acetylation and methylation was not clearly established.

Furthermore, ligand-dependent activation of FXR was reported to promote the recruitment of protein arginine methyltransferase 1 (PRMT1) and histone H4 methylation to the promoter of BSEP and SHP genes [40].

## 4. Targeting Chromatin and Chromatin-Associated Factors: New Opportunities for Handling Metabolic Disorders

A variety of epigenetic changes occur during cancer development [41], and particularly, disruption of HAT or HDAC activity can be associated with the development of cancer [42]. Therefore, the possibility to target tumor cells through epigenetic approaches, in particular by modulating HDAC activity, has emerged as a promising pharmacological tool for cancer therapy and several HDAC inhibitors have entered clinical trials in the last few years [43].

In addition to anticancer therapy, the detailed analysis of the epigenetic mechanisms responsible for the regulation of cholesterol catabolism described in the previous sections, provides the molecular rationale to envisage that HDACs, in particular HDAC7, could be targeted also to modulate cholesterol homeostasis.

Furthermore, HDACs could also be targeted for the treatment of metabolic disorders such as insulin resistance and diabetes, on the basis of several evidences. First of all, genome wide scans for both type 1 and type 2 loci suggested that HDACs may be implicated in the pathogenesis of diabetes [44]. Furthermore, several studies demonstrated that HDACs, especially HDAC4 and 5, play a pivotal role in regulating the expression of metabolic genes in skeletal muscle [45,46,47].

Finally, emerging evidences indicate that SIRT1, owing to its deacetylating activity on a wide range of substrate proteins and to other deacetylase-independent effects, would represent a potential target in several diseases, from cancer to metabolic disorders.

### 4.1. Histone Deacetylase Inhibitors

In recent years several natural compounds have been added to the long list of HDAC inhibitors that comprises chemicals belonging to different classes, several of which have been approved by Government Agencies for the treatment of malignancies.

The beneficial effects of dietary isothiocyanates and allyl sulfides on human health, and in particular their cancer chemopreventive effects, have been known for a long time [48,49], however, only recently their positive actions have been linked with epigenetic mechanism. A major understanding of HDAC active site structure and of the molecular features required to inhibit HDAC catalytic activity, in particular the presence of a spacer “arm” that allows the entry into the catalytic pocket and of a functional group interacting with the zinc cation, led to the reconsideration of the biological activity of several natural compounds. 

Sulforaphane (SFN) is one of the most characterized isothiocyanates found in vegetables. It derives from the glucosinolate glucoraphanin present in cruciferous vegetables, such as broccoli and broccoli sprouts. Like other isothiocyanates, it is metabolized via the mercapturic acid pathway to active metabolites, among which SFN-cysteine displays a good fit for HDAC active site according to computer modeling predictions [50]. Indeed, the effects of SFN in *in vitro* and *in vivo* systems are associated with increased global histone acetylation [51,52,53].

Allyl compounds are garlic components comprising diallyl disulfide and S-allyl mercaptocysteine, which are both converted into the active metabolite allyl mercaptane. Docking simulation revealed a good fit between allyl mercaptane and HDAC active site, consistent with accumulation of acetylated histones and growth arrest in cancer cells treated with the active metabolite at micromolar concentrations [54].

Finally, sodium butyrate should also be included in the list of naturally occurring HDAC inhibitors since it is generated during the fermentation of dietary fibers in the large intestine [55].

### 4.2. Effects of Histone Deacetylase Inhibitors on Cholesterol Metabolism

Based on our findings reported above, we hypothesized that targeting HDACs with an appropriate inhibitor may release *Cyp7a1* from the physiological repression caused by bile acids and, consequently, *in vivo* may also decrease blood cholesterol as a result of its enhanced conversion to bile acids. First of all, we could demonstrate that treatment of cultured liver cells with two chemically unrelated HDAC inhibitors, valproic acid and trichostatin A, indeed causes derepression of *CYP7A1* [38]. Next, we tested the effects of HDAC inhibitors on cholesterol metabolism in *Ldl-r*^-/-^ mice, an animal model of genetic hypercholesterolemia caused by a defect of the low-density lipoprotein receptor. We found that both inhibitors dramatically elevate *Cyp7a1* mRNA levels in *Ldl-r*^-/-^ mice [38]. Consistent with this, also fecal bile acid excretion, an index of bile acid synthesis, increases significantly in treated mice. But most interestingly, we also observed a parallel decrease of plasma cholesterol. The analysis of the expression profile of other key genes involved in cholesterol homeostasis showed no changes due to the treatment, thus confirming that the effects of HDAC inhibitors on plasma cholesterol levels in hypercholesterolemic mice are most likely secondary to their action on *Cyp7a1*.

### 4.3. Effects of Histone Deacetylase Inhibitors on Energy Metabolism

In addition to the effects on cholesterol and bile acid metabolism mentioned above, we also found that HDAC inhibitors affect in some way energy metabolism. In fact, we found that treated mice display decreased plasma triglycerides and no weight gain, despite increased food consumption (Mitro, Galmozzi, Gilardi, Godio, Scotti, Caruso, De Fabiani, Crestani, unpublished observations). A recent study provided further evidence supporting the involvement of HDACs in energy expenditure. In fact, Gao *et al*. reported that sodium butyrate, a dietary component found in cheese and butter and also produced in the large intestine after fermentation of dietary fibers, improves metabolic dysfunction in mice fed a high-fat diet [56]. In particular, supplementation with sodium butyrate prevents diet-induced obesity, increase energy expenditure, and improves insulin sensitivity. These metabolic changes reflect beneficial effects of sodium butyrate on brown adipose tissue and skeletal muscle since treated mice show increased adaptive thermogenesis, and a higher number of oxidative fibers, coupled with enhanced fatty acid oxidation and mitochondrial function, in skeletal muscles.

### 4.4. Effects of SIRT Activators on Energy Metabolism

A detailed discussion on the multiple downstream effects following the modulation of SIRT1 activity is far beyond the scope of this review. Therefore, here we will simply review the involvement of SIRT1 activation in the control of energy metabolism.

In 2003, Howitz *et al*. reported for the first time that small molecules, such as the natural polyphenol resveratrol, can extend the lifespan of *Saccharomyces cerevisiae* in a Sir2-dependent manner [57]. Subsequent studies in animal models of insulin resistance and high-calorie diet showed that resveratrol administration ameliorates the metabolic derangements observed in these mice [58,59]. In particular, resveratrol attenuates adipogenesis and fat storage in white adipose tissue and, at the same time, improves mitochondrial activity and thermogenesis of brown adipose tissue. Resveratrol also affects insulin and glucose homeostasis since it increases insulin secretion from the pancreas and improves insulin sensitivity in the skeletal muscle, accompanied by enhanced mitochondrial activity and fatty acid oxidation. These metabolic effects are associated to activation of AMP-activated kinase and PGC-1α. In particular, this latter effect is due to SIRT1-dependent deacetylation [58]. Indeed, all these factors are strictly and coordinately linked. In fact, AMP-kinase, a sensor of the cellular energy status that is activated by a rise of AMP/ATP ratio, enhances SIRT1 activity due to increased NAD^+^ cellular levels and consequently causing deacetylation of PGC-1α and FOXO transcription factors [60].

Although the animal studies discussed above clearly indicated that the effects of resveratrol are SIRT1-dependent, the observation that this polyphenol also activates AMP-kinase, makes difficult to establish which the primary target of resveratrol is. In fact, resveratrol increases SIRT1 deacetylating activity toward synthetic substrates containing a fluorescent moiety, but appears to have no direct effect on physiological substrates [61,62].

Collectively, these evidences indicate that resveratrol can modulate gene transcription and, consequently, metabolic pathways through specific molecular mechanisms that are independent from its antioxidant properties. 

## 5. Final Considerations

The fasted-to-fed cycle is characterized by hormonal fluctuations and by variations in the circulating levels of other signaling molecules that, through the entero-hepatic circulation, connect the gut to the liver. Recent studies have allowed reconsidering the function of bile acids in this respect, and have demonstrated that they participate in the regulatory mechanisms responsible for the metabolic adaptation occurring during the fasted-to-fed cycle, modulating the transcription of genes that play a key role in the synthesis of bile acids and gluconeogenesis.

The metabolic adaptation to fasting and to calorie restriction is indeed very complex, and the intracellular decrease of energy substrates represents *per se* a signal that activates a program of metabolic adaptation involving SIRT1, AMP-kinase, and PGC-1α.

Emerging evidences indicate that nutritional-linked cues, such as bile acids themselves, regulate the transcription of these genes by affecting the structure of chromatin. Histone deacetylases are deeply involved in chromatin dynamics and inhibition of their activity represents a way to regulate cholesterol homeostasis and energy metabolism.

Some nutritional compounds display the ability to affect the activity of chromatin modifying enzymes, *i.e.*, by inhibiting HDACs or activating sirtuins, thus participating in the epigenetic control of metabolic pathways. Therefore dietary manipulation aimed at increasing the intake of these molecules may be beneficial in the management of metabolic disorders such as obesity, metabolic syndrome, and diabetes.

When dealing with the biological effects of nutrients or of specific compounds present in foods, the nutritional aspects should be carefully considered.

A first issue that may concern nutritionists is to establish whether the regular intake of certain foods (vegetables, fibers) is sufficient to provide the amount of specific compounds necessary to elicit the biological effects reported in experimental studies. Indeed, the animal studies documenting the beneficial effects of naturally occurring isothiocyanates or resveratrol were performed administering diets supplemented with high amounts (from few to several hundred mg/kg of body weight) of pure compounds. It is questionable that such high amounts of individual compounds can be reached in humans simply by eating foods enriched in these molecules. Furthermore, concentration, composition and stability of these compounds in foods may vary due to multiple reasons, e.g. cultivars, ripening period, preservation conditions, cooking. For example, three-day-old broccoli and cauliflower sprouts contain 10-100 times higher levels of glucoraphanin than the corresponding mature plants.

One way to overcome this problem would be to prepare special foods or nutritional supplements enriched in these compounds. Resveratrol is present at high concentrations in the skins of red grapes and in the Japanese knotweed, a non-edible plant native to Eastern Asia that has become invasive in Western countries. Indeed, resveratrol tablets, prepared from plant sources, are advertised and marketed as nutritional supplement or “phytonutrient”. However, regarding its use as SIRT activator and therefore in the management of metabolic disorders, more studies are needed to establish whether beneficial effects ascribable to resveratrol can be obtained through the consumption of red grapes, red grape foods or directly by supplementing the diet with polyphenolic extracts [63].

In most cases natural compounds exhibit several biological activities contributing to the final effects in a whole biological system. Limiting the analysis to compounds acting through epigenetic mechanisms, we mention the example of isoflavones, abundant in plants of the Leguminosae family. These compounds were recently reported to induce mitochondrial biogenesis in cellular models, through SIRT and PGC-1α, in an estrogen-independent manner [64]. However, isoflavones have been known for many years for their phytoestrogenic activities, therefore it is expected that the *in vivo* effects following the consumption of isoflavone-rich foods, will be the result of their multiple actions.

 In conclusion, we expect that a deeper understanding of the epigenetic mechanisms involved in the control of metabolic pathways will allow to disclose novel effects associated either with dietary regimens (e.g., calorie restriction) or with consumption of specific nutrients (e.g., vegetables, fibers). Experimental studies, coupled with a thorough evaluation of nutritional aspects, are essential to assess the benefits of nutrition on human health through an “evidence-based” approach.
